# A method for ultrafast tissue clearing that preserves fluorescence for multimodal and longitudinal brain imaging

**DOI:** 10.1186/s12915-022-01275-6

**Published:** 2022-03-29

**Authors:** Qing-Hong Shan, Xin-Ya Qin, Nan Zhou, Chuan Huang, Yu Wang, Peng Chen, Jiang-Ning Zhou

**Affiliations:** 1 Chinese Academy of Science Key Laboratory of Brain Function and Diseases, School of Life Sciences, Division of Life Sciences and Medicine, University of Science and Technology of China, Hefei, 230026 China; 2 Department of Neurosurgery, Huashan Hospital, Fudan University, Middle Urumqi Road 12, Shanghai, 200040 China; 3 Center for Excellence in Brain Science and Intelligence Technology, Chinese Academy of Sciences, Shanghai, 200031 China

**Keywords:** Ultrafast tissue clearing, Three-dimensional visualisation, Fluorescence enhancement, Neuro-circuit tracing, 3D histopathology imaging

## Abstract

**Background:**

Tissue-clearing techniques have recently been developed to make tissues transparent for three-dimensional (3D) imaging at different scales, including single-cell resolution. However, current tissue-clearing workflows have several disadvantages, including complex protocols, time-consuming application, and fluorescence quenching. Additionally, they can be used mainly for clearing larger-volume samples, preventing wide and easy applicability in conventional experimental approaches. In this study, we aimed to develop a versatile, fast, and convenient method for clearing thin and semi-thick samples, which can be used for three-dimensional imaging of experimental or even clinical samples.

**Results:**

We developed an alkaline solution (AKS) containing a combination of 2,2′-thiodiethanol (TDE), DMSO, D-sorbitol, and Tris for tissue clearing, as the alkaline environment is suitable for maintaining the fluorescence of most commonly used fluorescence protein GFP and its variants, and tested its clearing effect on samples from mice and human brains. We assessed the clearing speed, the preservation of fluorescence protein and dyes, and the imaging depth and quality. The results showed that AKS treatment rapidly cleared 300-μm-thick brain slices and 1-mm-thick slices from different organs within 5 min and 1 h, respectively. Moreover, AKS was compatible with a variety of fluorescence proteins and dyes. Most importantly, AKS enhanced the fluorescence of YFP, in contrast to the majority of existing tissue-clearing methods which reduce the fluorescence intensity of fluorescent proteins. Using AKS, we performed long-time high-resolution imaging of weak fluorescent protein-labelled tissues, long-distance fibre tracking, larger-scale 3D imaging and cell counting of the entire brain area, neural circuit tracing, 3D neuromorphic reconstruction, and 3D histopathology imaging.

**Conclusions:**

AKS can be used for simple and rapid clearing of samples from mice and human brains and is widely compatible with a variety of fluorescent dyes. Therefore, AKS has great potential to be used as a broad tissue-clearing reagent for biological optical imaging, especially for time-sensitive experiments.

**Supplementary Information:**

The online version contains supplementary material available at 10.1186/s12915-022-01275-6.

## Background

Biological tissue is a structural and functional complex formed by the organic combination of many cells at the three-dimensional (3D) level. Therefore, the study of tissue morphology is the basis for studying biological functions. Conventional morphological studies observe the number, distribution, and morphology of different types of cells using tissue sections [1, 2]. However, such studies suffer from tissue deformation, insufficient details, and difficulty in 3D reconstruction, all of which make it difficult to obtain the real 3D information of the examined tissue. Some sequence-slicing techniques, such as micro-optical sectioning tomography (MOST) and fluorescence MOST (fMOST) techniques, can provide micrometre-scale tomography of a centimetre-sized whole mouse brain by performing the imaging and sectioning simultaneously [3–5]. However, those methods require professional equipment and are not compatible with immunostaining, which limits their application.

With the development of imaging technology and tissue-clearing technology in recent years, researchers have been able to image mice from the single-cell level to the whole-body level by using different tissue-clearing technologies [6–9]. For example, SeeDB, CLARITY, CUBIC, ScaleS, and MACS can be used for clearing and 3D imaging of the whole brain [7, 9–13]. SeeDB2 can be used for large-scale high-resolution imaging [14]. FOCM can be used for ultrafast optical clearing [15], while iDISCO and CUBIC-HistoVIsion can be used for 3D immunostaining of the whole brain [16, 17]. PARS and PEGASOS can even be used for whole-body clearing [6, 8]. However, the current tissue-clearing technology is usually designed for clearing large-volume samples. It usually requires hydrogel embedding (CLARITY, PACT), delipidation with organic solvents (3DISCO, BABB) [18, 19], or a high concentration of detergents (CUBIC, SWITCH) [20, 21]. Moreover, the inappropriate environment with inevitable protein denaturation causes fluorescence quenching [6, 21–24]. Therefore, the current optical clearing methods have the disadvantages of considerable time consumption, complex protocols, and even fluorescence quenching.

To reduce the difficulty and fluorescence quenching and improve the efficiency of tissue clearing, we reported a one-step method for tissue clearing based on an alkaline solution (termed AKS). AKS is an alkaline aqueous solution consisting of Tris base, dimethyl sulfoxide (DMSO), 2,2′-thiodiethanol (TDE), and D-sorbitol. By simple immersion in AKS, 300-μm-thick brain sections were cleared within 5 min, and the fluorescence signals of tdTomato and YFP were significantly enhanced. Moreover, the reagent was also compatible with a variety of fluorescent dyes. Thus, AKS is a simple and effective protocol for 3D imaging of various types of fluorescently labelled biological tissues.

## Results

### Development of AKS for simple and rapid clearing

Biological tissues appear opaque due to the existence of a variety of substances with different optical properties. In biological tissues, water has a refractive index (RI) of 1.33, proteins have RI of above 1.44, and lipids have an RI above 1.45 [6, 9, 25, 26]. Therefore, the use of high-RI solutions to perform RI homogenisation is the key step in tissue clearing. Here, we designed a water-soluble tissue-clearing reagent with a high RI for rapid tissue clearing. The reagent was composed of 40% TDE, 20% DMSO, 20% sorbitol, and 0.5 M Tris. Its pH was 10.5, and RI was 1.47 at 25°C. TDE was chosen because of its ability to adjust the RI of the solution, and it was used for RI matching [27, 28]. DMSO has been shown to increase the permeability of tissues [29, 30]; D-sorbitol was used for its ability to preserve fluorescence and tissue-clearing properties [10]; and Tris was used to maintain the pH of the solution.

**Fig. 1:**
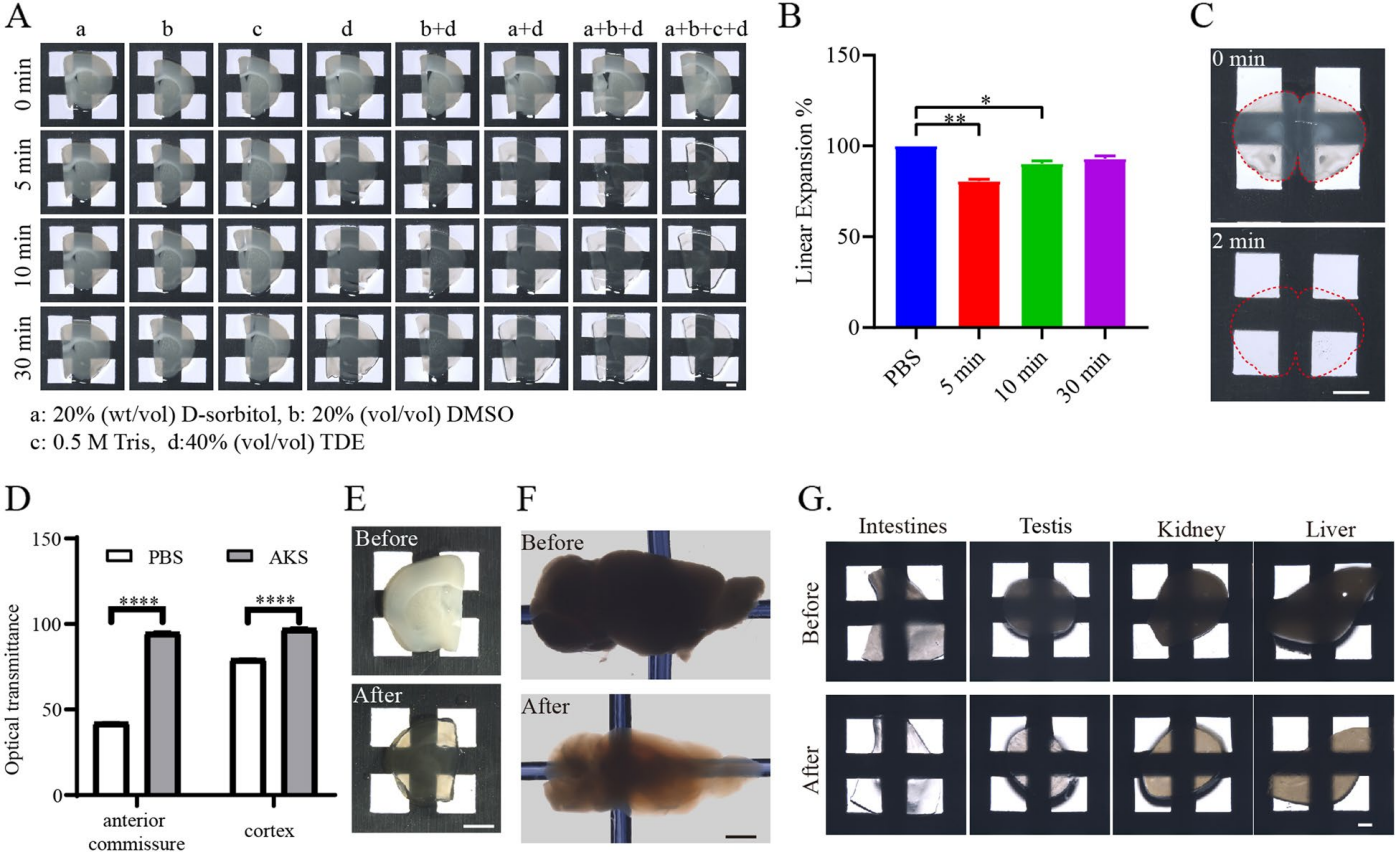
Ultrafast tissue clearing with AKS. **A** Transparency of 300-μm-thick slices before and after clearing with different components of AKS. a is 20% (wt/vol) D-sorbitol, b is 20% (vol/vol) DMSO, c is 0.5 M Tris, and d is 40% (vol/vol) TDE. Representative of *n* = 3 slices (scale bar: 1 mm). **B** Statistical data showing the sample morphology changes after AKS treatment at different time points. All values are presented as mean ± SEM and included in Additional file 11: Table S1. Statistical significance (*n* = 4, **p < 0.01) was assessed by a one-way ANOVA followed by Bonferroni’s multiple comparison tests. **C** Rapid clearing of 100-μm-thick brain slice within 2 min. Representative of *n* = 3 slices (scale bar: 2 mm). **D** Change in optical transmittance of different brain regions of 100-μm-thick slices before and after AKS treatment. All values are presented as mean ± SEM and included in Additional file 11: Table S2. Statistical significance (*n* = 4, ****p<0.0001) was assessed by paired *t*-test. **E** A 1-mm-thick brain slice before and after clearing with AKS for 1 h. Representative of *n* = 3 slices (scale bar: 2 mm). **F** Hemibrain of mice before and after clearing with AKS for 8 h. Representative of *n* = 3 hemibrains (scale bar: 2 mm). **G** The 1-mm-thick sections from different organs before and after clearing with AKS treatment for 1 h. Representative of *n* = 3 samples each tissue (scale bar: 1 mm)

To test the effects of different components of AKS on tissue transparency, we examined the effects of different components and their combinations on 300-μm-thick mouse brain slices. We found that any of these substances alone was not able to complete the clearing of the tissue, although 40% TDE (RI = 1.40) had a weak clearing effect (Fig. 1A). Furthermore, we observed that the combination of multiple components increased the clearing effect. We found that the combination of 20% DMSO in 40% TDE (RI = 1.43) or 20% sorbitol in 40% TDE (RI = 1.43) increased the effect of tissue clearing. The combination of 20% DMSO, 20% sorbitol, and 40% TDE (RI = 1.47) had a better clearing effect, indicating that the clearing effect was dependent on RI. The combination of 0.5 M Tris, 20% DMSO, 20% D-sorbitol, and 40% TDE (RI = 1.47) had the best clearing effect, with 300-μm-thick slices becoming transparent within 5 min (Fig. 1A). Tris is an amino alcohol compound, and its amino group can promote the tissue-clearing effect. We termed this combination of reagents as AKS. The linear expansion was 80.69 ± 1.04% (mean ± SEM) after 5 min of AKS treatment due to the high osmotic pressure of AKS. As the treatment time increased to 10 min and 30 min, the linear expansion is 90.36 ± 1.40% (mean ± SEM) and 93.11 ± 1.48% (mean ± SEM), respectively. The linear expansion did not differ significantly between 10 min and 30 min (*p* = 0.1131), indicating that after 10 min of AKS treatment the sample reached a stable state and preserved the morphology and clearing effect well (Fig. 1B). To test the clearing effect of AKS on thin tissue slices, 100-μm-thick brain slices were used. We found that the AKS treatment achieved full transparency of the 100-μm-thick brain slices within 2 min (Fig. 1C), and the light transmittance of the cortical area and the lipid-rich anterior commissure area significantly increased (Fig. 1D), indicating that AKS can be used for rapid clearing of thin slices, just like a mounting medium. Subsequently, we examined whether AKS can be used for clearing larger-volume samples. To that end, we used 1-mm-thick slices or hemibrains from mice. After the AKS treatment, 1-mm-thick brain slices became transparent within an hour (Fig. 1E), and the hemibrains became transparent within just 8 h (Fig. 1F), indicating that AKS can also be conveniently used for rapid clearing of large-volume samples. In addition, 1-mm-thick slices of the testis, kidney, liver, and intestines were used to examine whether AKS can be used for clearing peripheral organs. Our results showed that these tissues also appeared transparent after 1 h of AKS treatment, indicating that AKS can be used for rapid clearing of tissues from various peripheral organs (Fig. 1G). However, it is worth mentioning that AKS treatment induced rapid tissue clearing and did not have the decolorizing effect. Indeed, the pigment-rich liver and kidney tissues still had some opacity (Fig. 1G).

### Wide compatibility with fluorescent proteins and fluorescent dyes

**Fig. 2:**
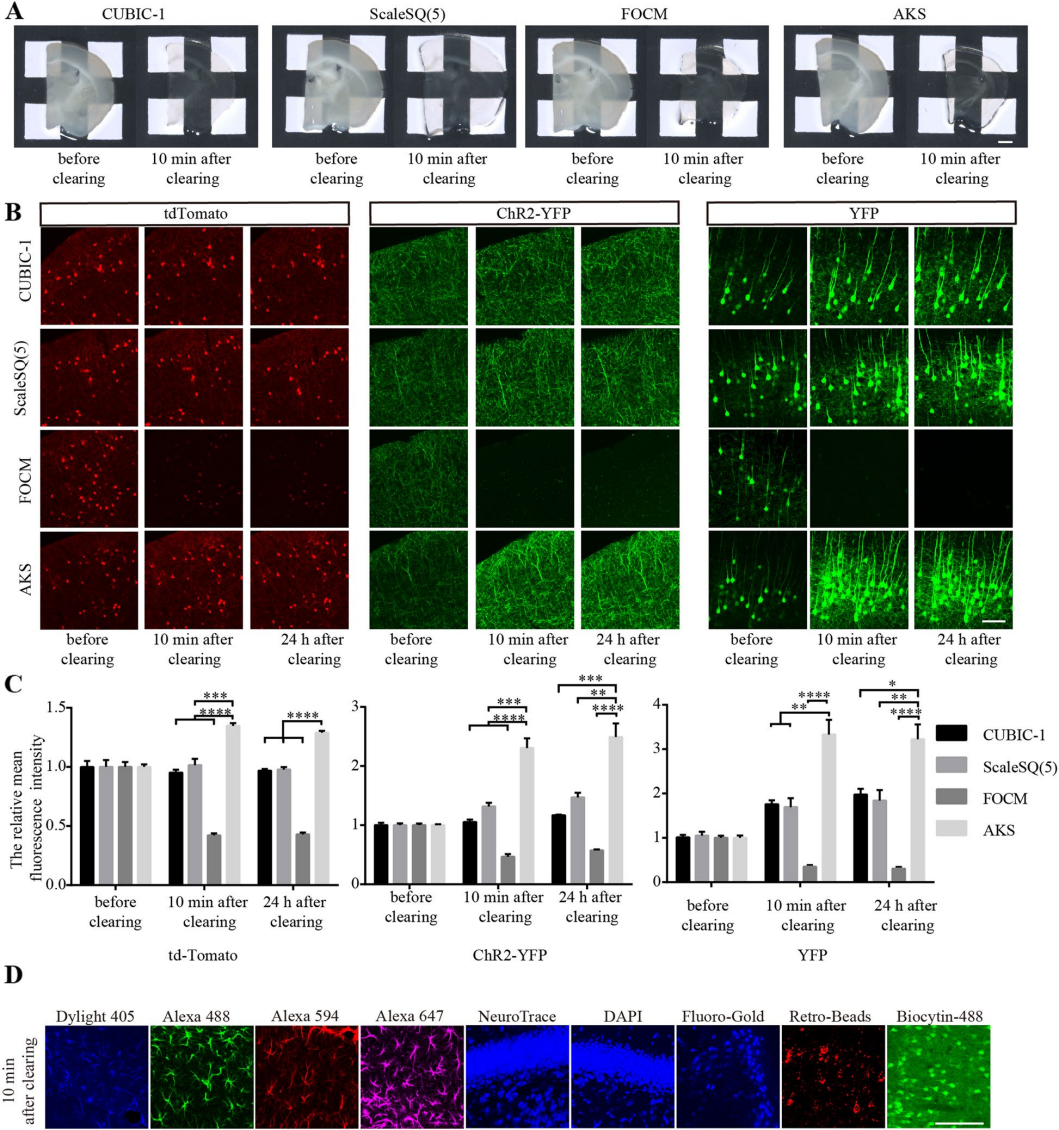
Fluorescence preservation after clearing. **A** Rapid clearing of 300-μm-thick slices with CUBIC-1, ScaleSQ(5), FOCM, and AKS. Representative of *n* = 3 slices (scale bar: 1 mm). **B** Preservation of fluorescent proteins under different reagent treatments. Representative of *n* = 3 slices each conditioning (scale bar: 100 μm). **C** Comparison of the normalised fluorescence of AKS-treated tissue (*n* = 3) with CUBIC-1 (*n* = 3), ScaleSQ(5) (*n* = 3), and FOCM-treated tissue (*n* = 3); the imaging parameters were the same and images were taken at different time periods. All values are presented as mean ± SEM and included in Additional file 11: Table S3. Statistical significance (*p < 0.05, **p < 0.01, ***p <0.001, ****p < 0.0001) was assessed by a one-way ANOVA followed by Bonferroni’s multiple comparison tests. **D** Preservation of fluorescent dyes after 10-min AKS treatment. Representative of *n* = 3 slices each conditioning (scale bar: 100 μm)

The most common application of tissue-clearing technology is in 3D imaging of fluorescent protein-labelled samples. Therefore, a variety of fluorescent proteins were used for testing the fluorescence signal preservation after the AKS treatment. Here, *CRH-cre;Ai32* mice (B6(Cg)-Crh^tm1(cre)Zjh^/J mice crossed with B6;129S-Gt (ROSA)26Sor^tm32(CAG-COP4*H134R/EYFP)Hze^/J mice) were used to express membrane-localised ChR2-EYFP [31, 32]. Thy1-YFP mice (B6.Cg-Tg (Thy1-YFP)HJrs/J) were used to express YFP in neuronal cytoplasm [33], and *CRH-cre;Ai14* mice (B6(Cg)-Crh^tm1(cre)Zjh^/J mice crossed with B6.Cg-Gt (ROSA)26Sor^tm14(CAG-tdTomato)Hze^/J mice) were used to express tdTomato [34]. To compare the fluorescence preservation by AKS and other rapid tissue-clearing methods, we selected CUBIC-1, ScaleSQ (5), and FOCM because they had been previously used for rapid tissue clearing [9, 10, 15]. To verify the rapid clearing ability of these methods, 300-μm-thick brain slices were immersed in CUBIC-1, ScaleSQ (5), FOCM, and AKS for 10 min at room temperature. We found that all of these reagents cleared the tissue rapidly (Fig. 2A). We then tested the preservation of fluorescence after the treatment with these reagents. To reduce the interference from the tissue deformation on the fluorescence test during the clearing process, 100-μm-thick brain slices were embedded on an adhesion microscope slide, treated with various clearing reagents for 10 min, and cover-slipped with the same reagent that was used for clearing. The images were taken before clearing (in PBS) and 10 min or 24 h after clearing. We found that the fluorescence of tdTomato, ChR2-EYFP, and YFP cleared by CUBIC-1, ScaleSQ (5), and AKS was well preserved, while the FOCM treatment reduced the fluorescence of tdTomato, ChR2-EYFP, and YFP (Fig. 2B). Moreover, the fluorescence of tdTomato, ChR2-EYFP, and YFP after the AKS treatment was stronger than that after the other three methods, and even stronger than that before clearing (Fig. 2C). For example, the signal of YFP after the AKS treatment was more than three times higher than that before clearing, showing that AKS processing can preserve and even enhance the fluorescence of the tissue. At the same time, we found that the enhanced fluorescence was due to the increased fluorescence of the fluorescent protein (Additional file 1: Fig. S1).

In addition to using fluorescent proteins for labelling, various types of fluorescent dyes are also used to label biological samples. Thus, we examined whether AKS was compatible with general staining methods such as immunofluorescence and chemical dyes (Fig. 2D). Here, a primary antibody against glial fibrillary acidic protein (GFAP) was applied to label astrocytes in the hippocampus, and secondary antibodies conjugated with DyLight 405, Alexa Fluor 488, Alexa Fluor 594, and Alexa Fluor 647 were used for the testing. We found that the fluorescence signal was well preserved after 10 min of AKS treatment. DAPI and NeuroTrace are commonly used fluorescent dyes for counterstaining [35]. Here, we found that 10 min of AKS treatment also preserved their fluorescence. Fluoro-Gold and RetroBeads are commonly used for retrograde tracing [36]; we found that the 10 min of AKS treatment well preserved their fluorescence signal. Biocytin-488 is a commonly used dye for cell morphology labelling in electrophysiological experiments; we found that AKS was also compatible with biocytin-488. Therefore, we concluded that AKS can be widely used in experiments with a variety of fluorescent dye-labelled tissues.

To test the stability of the AKS-cleared tissue over time, we examined the effect of long-term processing of AKS on a variety of fluorescent proteins and fluorescent dye-labelled samples. We found that under the conditions of imaging 10 min after clearing with AKS, most of the fluorescent signals were well preserved and only the fluorescence of Dylight405 was weakened. Also, even after 2 weeks of storage in AKS solution, most of the fluorescent signals were well preserved, indicating that tissue cleared with AKS was very stable (Additional file 2: Fig. S2).

### AKS enables high-resolution and long-term imaging of weakly fluorescent-labelled samples

**Fig. 3:**
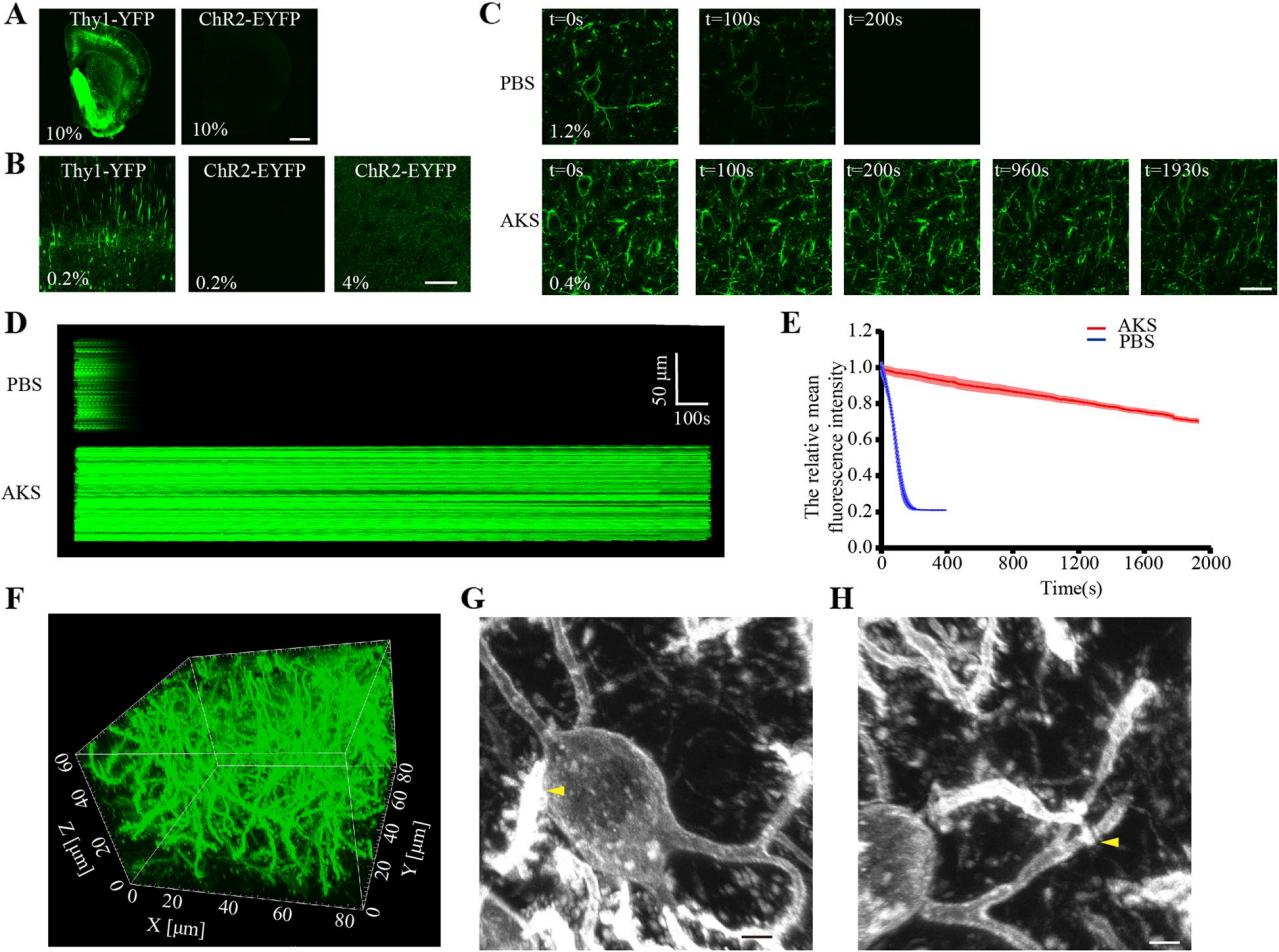
Long-term high-resolution images of weak fluorescent protein-labelled tissue. **A**, **B** Image of YFP- and ChR2-YFP-labelled brain slices; the numbers in the images indicate the power of the laser used. Representative of *n* = 3 slices each conditioning. **A** is a large-field view and **B** is the image of the cortex (scale bars: 1 mm in **A** and 200 μm in **B**). **C** Representative images of high-resolution time-series imaging of ChR2-YPF-labelled tissue before and after AKS treatment. The numbers in the images indicate the power of the laser used. Representative of *n* = 3 slices each conditioning (scale bar: 20 μm). **D** Time-stack images showing the photobleaching effect before and after AKS treatment around imaging time of **C**. Representative of *n* = 3 slices each conditioning. **E** Analysis of the relative mean fluorescence intensity around time-series imaging of **C** (*n* = 3 slices each conditioning). All values are included in Additional file 11: Table S4. **F** 3D high-resolution imaging of the ChR2-YFP-labelled CRH neurons in the central amygdala. Representative of *n* = 2 samples. **G** Image cropped from **F**, showing how the dendrites of CRH neurons connect to the soma of CRH neurons in the central amygdala, indicated by a yellow arrow (scale bar: 2 μm). **H** Image cropped from **F**, showing the dendrites of CRH neurons connecting to dendrites of CRH neurons in the central amygdala, indicated by a yellow arrow (scale bar: 2 μm)

Tissues from Thy1-YFP or Thy1-GFP mice have generally been used to develop most tissue-clearing methods [7, 11, 15]. However, in certain experiments, a problem in clearing and 3D imaging of weakly fluorescent-labelled samples was encountered. Given that AKS achieved extremely fast optical clearing and fluorescence preservation, we tried to perform clearing and 3D imaging of weakly fluorescent protein-labelled samples by AKS treatment. We first compared the fluorescence intensity of Thy1-YFP and *CRH-cre;Ai32* mice. At the same excitation intensity, the fluorescence signal of Thy1-YFP brain slices was very strong and clearly observed, in contrast to the ChR2-EYFP-labelled brain slices (Fig. 3A), indicating that the signal of ChR2-EYFP was weaker than that of Thy1-YFP. Next, we found that even when the excitation intensity was increased by 20 times, the signal of ChR2-YFP was still very weak (Fig. 3B). This indicates that larger excitation intensity is required for samples with weak fluorescent protein labels, but that would increase the possibility of photobleaching. To test for the photobleaching effect, *CRH-cre;Ai32* mice were imaged through time-series imaging with LSCM (Olympus FV1200MPE with PLAPON60X objective lens). Before AKS clearing, the tissues needed three times higher excitation light intensity to obtain the same signal intensity as after the AKS treatment (Fig. 3C). Before the AKS treatment, obvious photobleaching occurred after only 100 s of imaging, and the fluorescence signal was almost completely bleached after 200 s (Fig. 3C–E). However, after AKS treatment, only weak excitation light was needed to achieve a satisfactory signal. After 200 s of continuous imaging, the signal was clearly visible, and even after 30 min of continuous imaging, the signal was only reduced by about 30% (Fig. 3E). These data indicate that the AKS treatment can significantly improve the imaging time and quality of the weakly fluorescent-labelled samples. To explore whether the long-term observation after AKS treatment was due to the use of a lower intensity laser or because the AKS treatment may have an anti-quenching effect, we tested the photobleaching of ChR2-EYFP under the same laser intensity. We found that regardless of whether it was processed by AKS or not, the fluorescence of ChR2-EYFP was quenched rapidly, indicating that the long-term observation after AKS treatment was caused by the use of low laser intensity (Additional file 3: Fig. S3). To test whether the AKS treatment can be used for high-resolution 3D imaging, the slices from *CRH-cre;Ai32* mice containing the central amygdala were used for imaging. We found that after clearing with AKS, weakly fluorescence-labelled samples could be subjected to high-resolution 3D imaging. The fluorescence signal was very uniform throughout the 50-μm imaging depth (Fig. 3F). At the same time, the internal structures of the tissues such as dendrites and dendritic spines were clearly visible (Fig. 3G). The connections between dendrites and cell bodies and those between dendrites and dendrites were also clearly visible (Fig. 3G, H; Additional file 7: Movie S1), indicating that AKS can be used for long-term high-resolution 3D imaging of weakly fluorescence-labelled samples.

### AKS enables long-range fibre tracking and larger-scale imaging and analysis

**Fig. 4:**
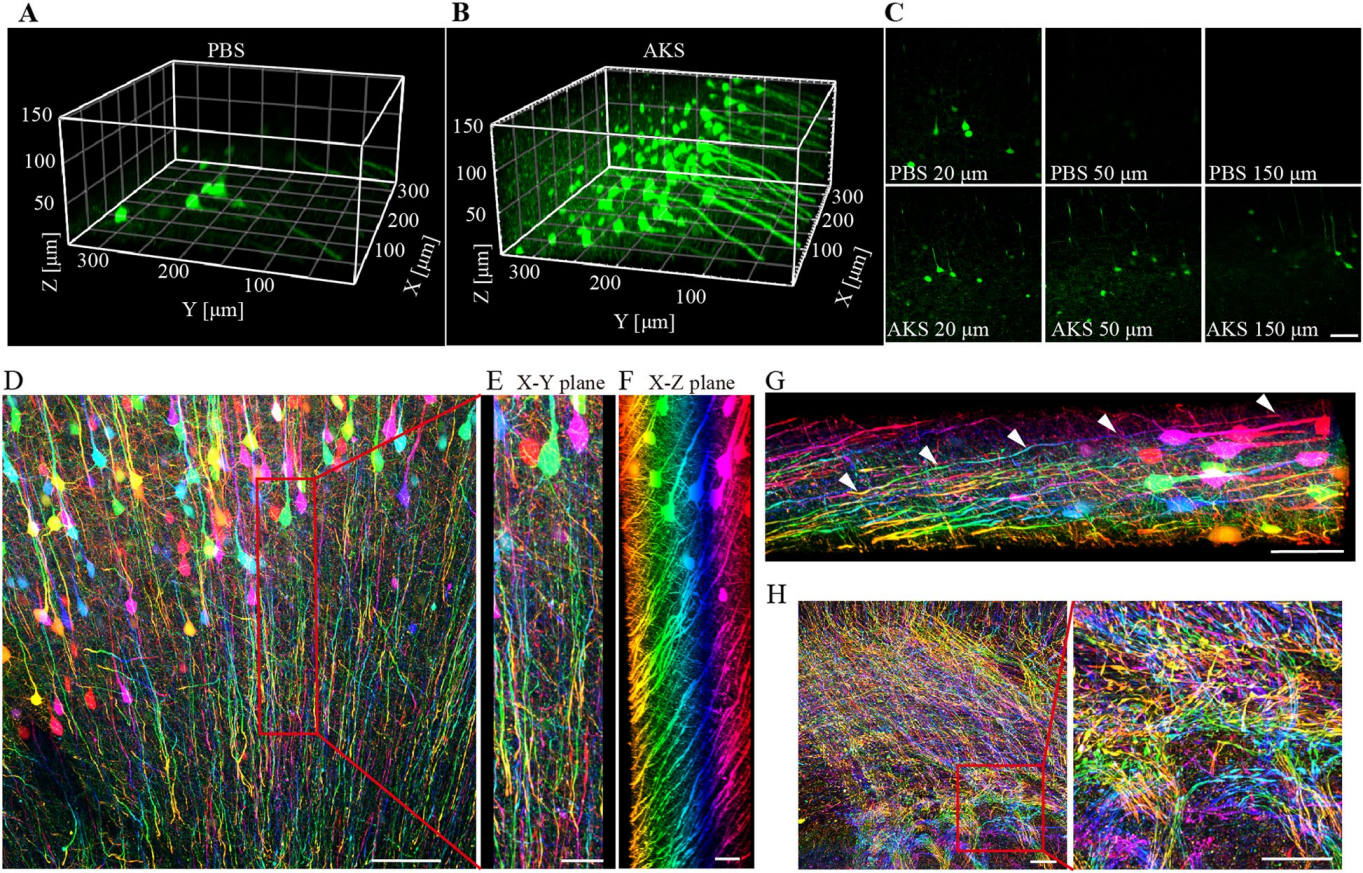
Nerve fibre tracking with AKS-cleared Thy1-YFP mice. **A**–**C** 3D imaging of 150-μm-thick slices from Thy1-YFP mice before and after AKS treatment. **A** 3D render of YFP imaged before AKS treatment. **B** 3D render of YFP imaged after AKS treatment. **C** XY plane of YFP signal at different image depths before and after AKS treatment. Representative of *n* = 3 slices each conditioning (scale bar: **C**, 100 μm). **D**–**F** 3D imaging of neurons and nerve fibres in AKS-cleared cortex. Representative of *n* = 3 slices (scale bars: **D**, 150 μm; **E** and **F**, 50 μm). **G** Long-range tracing of a single nerve fibre (scale bars: 100 μm). **H** Imaging of nerve fibres in the corpus callosum and striatum. Representative of *n* = 3 slices (scale bars: left, 100 μm; right, 100 μm)

Different brain regions are connected by long-distance nerve fibres. Therefore, studying the distribution and direction of nerve fibres is very important to understand the connections between different regions of the nervous system. Considering that YFP can nicely label the cell body and fibre morphology of neurons in Thy1-YFP-H mouse brain [37], slices from Thy1-YFP mouse brains were used to examine whether AKS could be used for fibre tracking. First, we examined whether AKS treatment can increase the image depth, and 150-μm-thick slices from Thy1-YFP brains were used for testing. We found that the slices without AKS treatment were only clearly visible at 20 μm thickness using a confocal microscope. In contrast, after AKS treatment, the entire 150-μm-thick slices could be clearly imaged (Fig. 4A–C), indicating that the AKS treatment can be used for 3D imaging of thicker samples. Subsequently, we performed three-dimensional imaging on the 300-μm-thick slices to track the nerve fibres. In order to show the spatial position of the fibres more clearly, we performed depth coding on the image. We found that after AKS treatment, the cell bodies and fibres of the neurons were clearly visible (Fig. 4D–F), and 800-μm-long nerve fibres could be tracked continuously (Fig. 4G). In addition to tracking individual nerve fibres, we also tried to track fibre bundles. We imaged the corpus callosum and striatum, which are rich in nerve fibre bundles, and clearly observed how the fibres from the cortex converged in the corpus callosum (Fig. 4H).

**Fig. 5:**
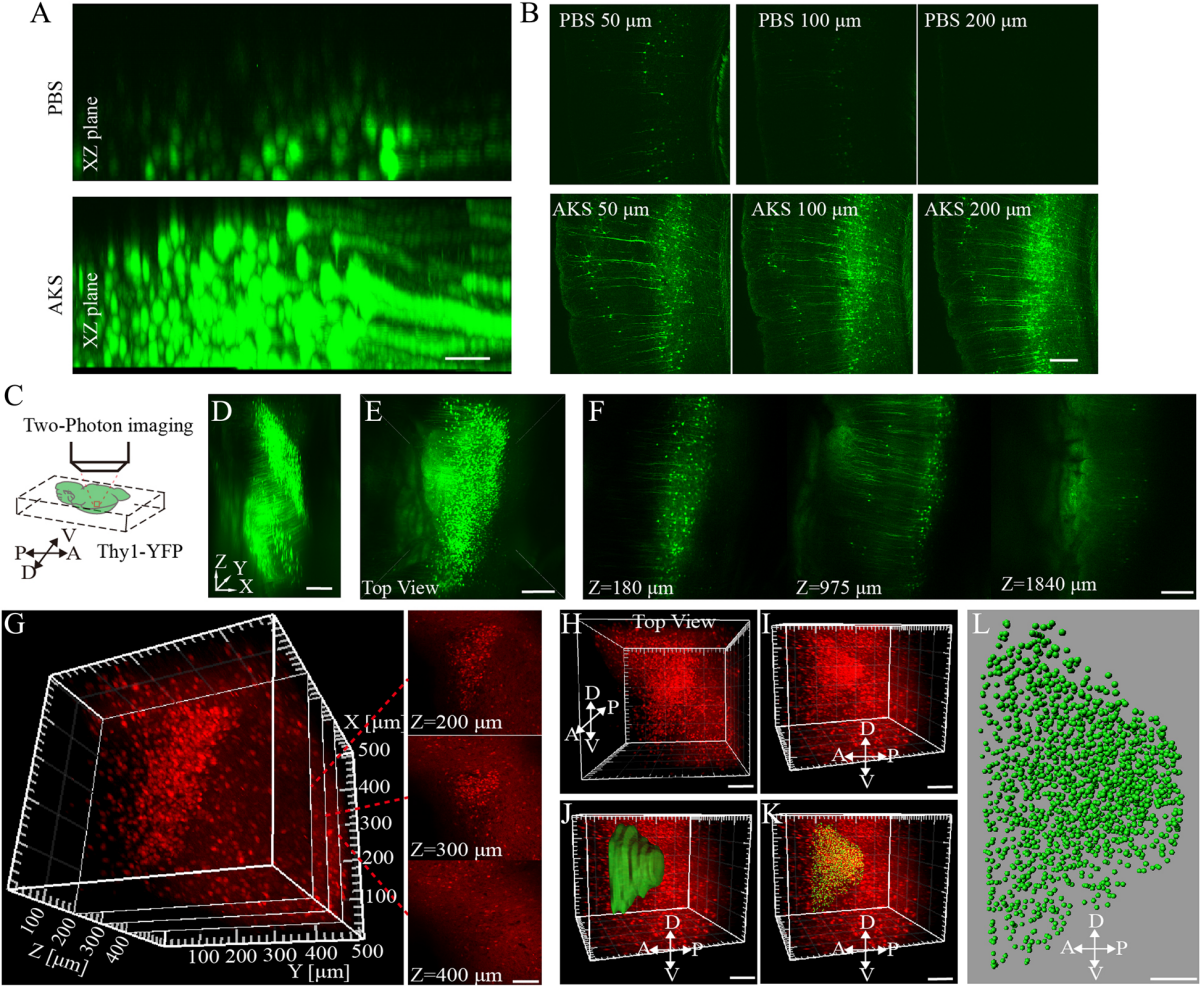
Large-volume imaging of AKS-cleared tissue using two-photon microscopy imaging. **A**, **B** 3D imaging of 150-μm-thick slice from Thy1-YFP mice before and after AKS treatment. **A** XZ plane showing YFP signal at different imaging depths before and after AKS treatment. **B** XY plane of YFP signal at different imaging depths before and after AKS treatment. Representative of *n* = 3 slices each conditioning (scale bar: **A**, 50 μm; **B**, 200 μm). **C**–**F** Imaging of the cortex of AKS-cleared hemibrain of Thy1-YFP mice. Representative of *n* = 2 hemibrain. **C** The imaging diagram. **D** 3D reconstruction of YFP-labelled neurons in the cortex (scale bars: 300 μm). **E** Top view of the 3D reconstruction (scale bars: 300 μm). **F** Optical sections of AKS-cleared Thy1-YFP mouse brain at various depths (scale bars: 200 μm). **G**–**K** 3D imaging and analysis of the tdTomato-labelled CRH neurons in *CRH-cre;Ai14* mice PVN. Representative of *n* = 2 slices. **G** Distribution of CRH neurons in different locations of the hypothalamus (scale bars: 100 μm). **H** Top view of 3D reconstructed CRH neurons in PVN (scale bars: 100 μm). **I** Side view of the 3D reconstructed CRH neurons in PVN (scale bars: 100 μm). **J**, **K** Reconstruction of the surface of the PVN area **(J)** and the location of CRH neurons **(K)** in PVN (scale bar: 100 μm). **L** Reconstructed 3D distribution of CRH neurons in PVN (scale bar: 50 μm)

In addition to using a confocal microscope to perform 3D imaging of the AKS-cleared tissue, we also applied two-photon microscopy to perform 3D imaging on the AKS-cleared tissue. We used 200-μm-thick slices from Thy1-YFP brains for testing. The results showed that the imaging depth was only about 50 μm without AKS treatment, whereas the entire 200-μm imaging range was clearly imaged after AKS treatment (Fig. 5A, B). These results indicated that AKS treatment could also increase the imaging depth when using two-photon microscopy. Subsequently, two-photon microscopy was used to image tissues from the liver, kidney, testis, and small intestine before and after AKS treatment. We found that AKS treatment also increased the imaging depth of these tissues (Additional file 4: Fig. S4). These results indicate that AKS treatment can be used for 3D imaging of the brain and peripheral tissues. To determine whether AKS treatment can be used to image large samples, we imaged the cortex of the hemibrains of Thy1-YFP-H mice treated with AKS for 8 h. We found that neurons were clearly visible through the 2-mm imaging range (Fig. 5C–F), indicating that AKS can be used for fast 3D imaging of large-volume samples.

Corticotropin-releasing factor (CRH), which is released from the paraventricular nucleus (PVN) of the hypothalamus, is essential for mediating stress response by activating the hypothalamus–pituitary–adrenal axis [38]; however, the complete 3D distribution of CRH neurons in PVN has rarely been reported. Through 3D fluorescence reconstructions with AKS, we were able to acquire a more complete signal distribution in a certain area; thus, we used AKS to observe the spatial distribution of CRH neurons in PVN. We cleared 1-mm-thick *CRH-cre;Ai14* mouse brain slices with AKS for 1 h and imaged them by a two-photon microscope. CRH neurons in PVN were mainly distributed in the imaging range of 100–400 μm (Fig. 5G), and the 3D distribution of CRH neurons in the whole PVN was also clearly seen (Fig. 5H, I). Imaris software was used for automatic identification and counting of CRH neurons in the PVN region (Fig. 5J, K; Additional file 8: Movie S2), resulting in 1523 neurons labelled with tdTomato in a half of the PVN region of a mouse (Fig. 5L).

### AKS enables spatial co-location analysis and 3D reconstruction of neural morphology

**Fig. 6:**
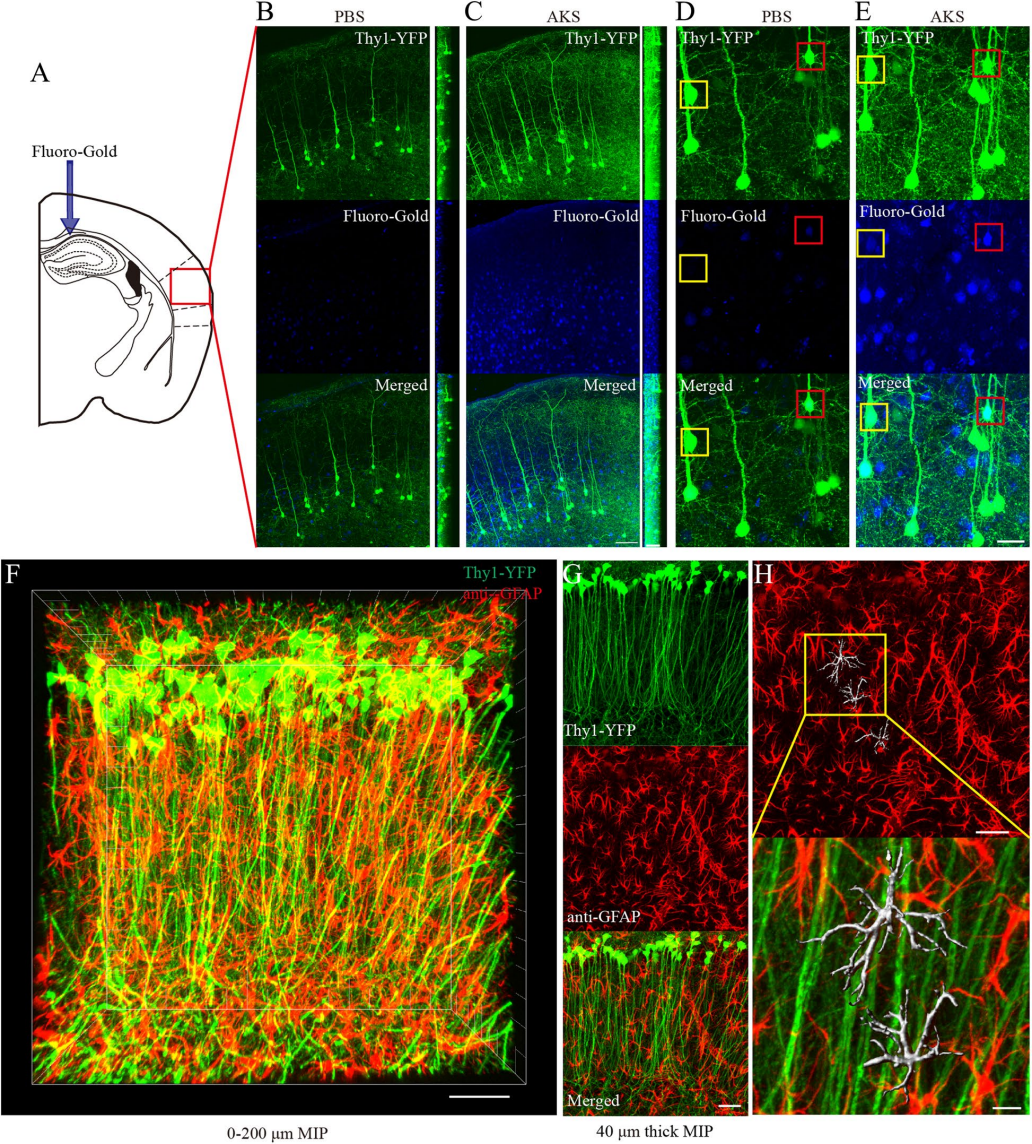
Spatial co-location analysis and reconstruction of neural morphology in 3D after AKS clearing. **A**–**E** Visualisation of spatial co-location of Fluoro-Gold- and YFP-labelled neurons in the cortex. Representative of *n* = 3 slices each conditioning. **A** Injection of Fluoro-Gold into Thy1-YFP mice corpus callosum. **B**, **C** 3D visualisation of Fluoro-Gold- and YFP-labelled neurons before and after AKS clearing (scale bars: left, XY view, 80 μm; right, XZ view, 50 μm). **D**, **E** 3D visualisation of Fluoro-Gold and YFP co-labelled neurons before and after AKS clearing, the enlarged view of the image is obtained from **B** and **C**, and the maximum intensity projection (MIP) of the 3D image is shown (scale bars: 30 μm). **F**–**H** 3D morphological reconstruction of astrocytes in the hippocampus. Representative of *n* = 3 slices. **F** 3D immunostaining of 300-μm-thick Thy1-YFP-H mouse slices with GFAP antibody; maximum intensity projection (MIP) image is shown (scale bars: 50 μm). **G** 40-μm-thick data cropped from **F** (scale bars: 40 μm). **H** 3D reconstruction of astrocytes inside the hippocampus (scale bars: above, 40 μm; below, 10 μm)

A variety of labelling methods are often used in combination for the labelling of biological tissues. Given that AKS was compatible with a variety of fluorescent proteins and fluorescent dyes, we examined whether AKS could be used to image samples labelled with both fluorescent protein and fluorescent dye. The corpus callosum of Thy1-YFP-H mice was injected with Fluoro-Gold for retrograde tracing and cut into 100-μm-thick slices for AKS treatment and analysis (Fig. 6A). Before the AKS treatment, the YFP signals were visible throughout the 100-μm imaging range, but the signal of Fluoro-Gold was only observed at the surface (Fig. 6B). We then treated these slices with AKS for 5 min and performed imaging with the same parameters. We found that the YFP signal and Fluoro-Gold signal were very uniform and clear throughout the imaging range (Fig. 6C). In addition, some Fluoro-Gold-labelled cells were very weak or even undetectable before the AKS treatment (Fig. 6D); however, after the AKS treatment, Fluoro-Gold-labelled cells were clearly visible. Moreover, some of the co-location signals that could have not been detected before processing were now detectable (Fig. 6E), indicating that AKS processing increased the sensitivity to co-location detection.

Since immunostaining is a commonly used method for identifying specific types of cells, we combined AKS and immunostaining for 3D morphological reconstruction. We labelled astrocytes by immunostaining of 300-μm-thick Thy1-YFP-H mouse tissue with an anti-GFAP antibody and then cleared the sections using AKS. We found that the entire *z*-axis range of the brain slices could be clearly imaged after the AKS treatment, and astrocytes were uniformly labelled (Fig. 6F; Additional file 9: Movie S3). However, because the presence of too many astrocytes in the 300-μm-thick hippocampus slices affected the observation of single astrocytes, 40-μm-thick volumes of interest were cropped for morphology reconstruction. After cropping, the morphology of astrocytes and their connections with blood vessels were clearly visible (Fig. 6G). Subsequently, we performed 3D reconstruction of several astrocytes with Imaris software (Fig. 6H), which can be used for further 3D morphology analysis.

### AKS enables 3D histopathology imaging of tissues from the human brain

**Fig. 7:**
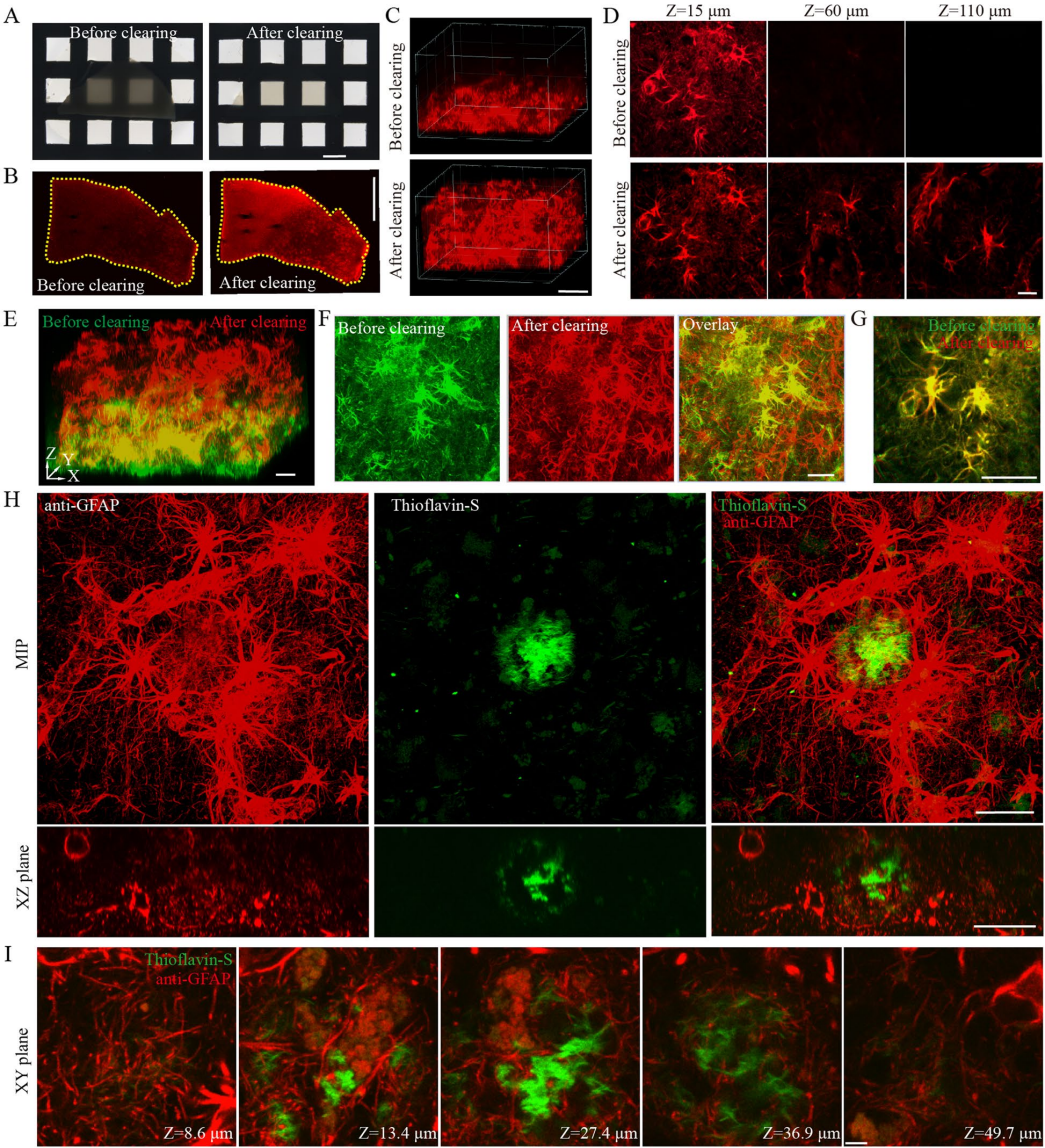
AKS treatment for clearing and imaging of the human brain samples. **A** Transparency of samples (300-μm-thick slice from formalin fixation for 2 years) before and after 10 min of AKS treatment for clearing. Representative of *n* = 3 slices (scale bar: 2 mm). **B** Sample size change before and after the AKS treatment. The slice is from fresh-frozen AD sample and immunofluorescence stained with GFAP. Representative of *n* = 3 slices (scale bar: 2 mm). **C**–**G** 3D imaging of GFAP-stained tissue before and after AKS clearing at the same imaging position and parameters. Representative of *n* = 3 slices. **C** 3D reconstruction of GFAP-stained tissue before and after AKS clearing (scale bar: 40 μm). **D** Selected single images at the depth of 15 μm, 60 μm, and 110 μm before and after the AKS clearing (scale bar: 15 μm). **E** Side view of the overlaid 3D-reconstructed GFAP-stained images before and after AKS clearing; note that more astrocytes are visible after clearing (scale bars: 30 μm). **F** Top view of the overlaid 3D-reconstructed GFAP-stained images before and after AKS clearing (scale bars: 30 μm). **G** A single image selected from **F**, showing the overlay of GFAP-labelled signal before and after AKS clearing (scale bars: 30 μm). **H** High-resolution 3D imaging and reconstruction of astrocytes and neuritic plaques. Representative of *n* = 3 slices (scale bars: 20 μm for maximum intensity projection (MIP) imaging and 30 μm for XZ plane imaging). **I** Selected single images at different imaging depths from **H**, showing the astrocyte fibres inside the neuritic plaques (scale bars: 5 μm)

After testing AKS in mice, we examined whether AKS can be used for rapid clearing of human brain samples. To that end, 300-μm-thick human brain slices kept in formalin for 2 years were used. We found that 10 min of AKS treatment significantly improved the transparency of the tissue (Fig. 7A) with slight deformation (Additional file 5: Fig. S5), suggesting that AKS can also be used for rapid clearing of human brain samples. Subsequently, we investigated whether the AKS treatment can be used for the observation of pathological samples. Since the preservation of tissue morphology and antigens is very important in pathological examination, 150-μm-thick sections from fresh-frozen Alzheimer’s disease (AD) samples were used for testing. First, we stained the samples with GFAP to label astrocytes, and then imaged the samples before and after clearing. We found that the morphological changes after AKS treatment were relatively small, and the images could be basically completely overlapped (Fig. 7B). Subsequently, we performed 3D imaging on the same area of the samples before and after clearing. We found that before AKS treatment, GFAP-labelled signals were only observed on the surface of the samples, and the fluorescence signal was weaker or even undetectable at a higher imaging depth. However, after AKS treatment, the GFAP-labelled signal was clearly observed in the entire 110-μm-thick imaging depth (Fig. 7C, D), showing that AKS treatment can improve the imaging depth and quality in human brain samples. Subsequently, we tried to overlay the astrocytes before and after AKS treatment at the 3D level to observe the morphological changes in the 3D level. After overlay, we clearly observed that AKS treatment increased the depth of imaging (Fig. 7E). The astrocytes imaged before and after clearing overlapped well at the 3D level (Fig. 7F), indicating that AKS treatment can preserve the 3D tissue structure and antigens. By overlapping a single plane from the Z-series before and after clearing, it was clearly observed that the morphological changes of astrocytes were very small, and the cells could be overlapped very closely (Fig. 7G). Since neuritic plaques in the brain are the neuropathological hallmark of AD, we applied AKS treatment to observe the 3D morphology of neuritic plaques and astrocytes in the human brain with AD. We stained the samples with GFAP immunofluorescence staining with thioflavin-S to label neuritic plaques. After the AKS treatment, the morphology of astrocytes and thioflavin-S-labelled neuritic plaques was well preserved, and astrocytes were clearly visible around the neuritic plaques (Fig. 7H). At a single plane, the GFAP fibres shuttling through the neuritic plaques were clearly identifiable (Fig. 7I; Additional file 10: Movie S4), indicating that AKS treatment can also be used for 3D observation of pathological samples.

## Discussion

To solve the problems of long processing time, complicated steps, and fluorescence quenching, all of which limit the application of tissue-clearing technology, here, we designed an alkaline tissue-clearing reagent AKS, a water-based ultrafast optical clearing method, containing Tris, D-sorbitol, DMSO, and TDE. We showed that our reagent was compatible with various labelling methods. AKS rapidly cleared tissues, accompanied with the enhancement of YFP fluorescence signals by a single and simple step immersion. AKS is nontoxic, preserves various fluorescence dye signals well, and can be used for 3D imaging very flexibly.

With the development of tissue-clearing technology in recent years, dozens of different types of reagents have been used. The role of these reagents can be mainly divided into five categories: dehydration, delipidation, hydration, decolourisation, and RI matching [39]. However, the commonly used tissue-clearing methods are more concerned with the effect on tissue transparency of larger-volume samples, and for the preservation of fluorescent protein labels, the current methods primarily focus on reducing fluorescence quenching. Given that low pH value, protein denaturation, and presence of free radicals in the solvent would lead to fluorescence quenching, an alkaline solution could well maintain the fluorescence of GFP and its variants [6, 21–23, 40–42]. Tris was chosen to create an alkaline environment suitable for GFP and its variants to actively increase the fluorescent protein signal. In addition, some tissue-clearing methods use toxic reagents, such as acrylamide or formamide [43], so attention is needed during processing. However, no toxic reagents are included in AKS. Most tissue-clearing methods, such as CLARITY, CUBIC, and 3DISCO, usually require multistep processing for tissue clearing. For example, they usually perform dehydration or delipidation first, and then RI matching is performed to complete tissue clearing. AKS uses the organic combination of various reagents to complete tissue clearing through a one-step process using RI matching. High RI water-miscible polar solvent TDE was used for the primary RI matching and dehydration reagent. The amphipathic molecule DMSO was used to increase the permeability of the tissue. Hydrophilic polyalcohol, sorbitol, was used to increase the clearing effect and fluorescence preservation. Tris, which belongs to amino alcohols, was used to create an alkaline environment to prevent fluorescence protein quench caused by protonation. AKS uses the synergistic effect of multiple reagents for rapid tissue clearing and good preservation of biomarkers, thereby preventing possible adverse effects of using a single reagent at a high concentration. Some tissue-clearing methods use alkaline solutions or amino alcohols to decolour the pigment-rich tissue (e.g. tissue from the liver or kidney) [9, 11]. AKS was used for rapid clearing and did not have the decolouring effect, on tissues rich in pigment (such as the liver and kidney), so it was expected that the light absorption would affect the clearing performance of AKS. However, we found that AKS treatment can be used for 3D imaging of 200-μm-thick slices from mouse liver and kidney without decolouration, indicating that the decolouration step may not always be necessary for thin slices. By comparing AKS with other existing methods in terms of versatility, clearing speed, ease of use, and fluorescence preservation (Table 1), AKS has the potential to be widely used in conventional 3D imaging.

**Table 1:** Comparison of AKS properties with other tissue-clearing methods. The table summarises the general properties of AKS and other major published clearing methods. In the column of “handling”, “Needs care” indicates that toxic reagents are used and need to be handled carefully

	Hydrogel embedding	Lipid remove	Step of operation	Fluorescent protein preservation	Human brain tissue compatible	Handling	Normal operating time	Reference
ASK	No	No	One step	Good, with strong YFP enhancement	Yes	Very easy	Minutes to hours	
CUBIC	No	Yes	One step or multistep	Good	Yes	Easy	Hours to days or weeks	[9, 44]
ScaleS	No	No	One step or multistep	Good	Yes	Easy	Hours to days or weeks	[10]
SeeDB	No	No	Multistep	Good	Not test	Easy	Several days	[13]
FOCM	No	No	One step	Good or major loss	Not test	Very easy	Minutes to hours	[15]
MACS	No	No	Multistep	Good	Not test	Easy	Hour to days	[11]
BABB	No	Yes	Multistep	Good or major loss	Not test	Needs care	Hour to days	[19]
3DISCO	No	Yes	Multistep	Major loss	Not test	Needs care	Hours to days	[18, 20]
PEGASOS	No	Yes	Multistep	Good	Yes	Easy	Hours to days	[6]
CLARITY	Yes	Yes	Multistep	Good	Yes	Needs care	Weeks	[7]
SWITCH	Yes	Yes	Multistep	Major loss	Yes	Needs care	Weeks	[45]
PACT	Yes	Yes	Multistep	Moderate loss	Yes	Needs care	Weeks	[8, 10]

Although the clearing speed is important for the tissue-clearing method, in most cases, it is even more important to preserve biological information inside the sample. In our experiment, similar to previous reports, ScaleSQ (5), CUBIC-1, and FOCM rapidly cleared the thin samples, but the fluorescence of tdTomato and YFP was usually lower than when treated with AKS, indicating better preservation of biological information with the AKS treatment. Thus, the AKS treatment is suitable for experiments needing high signal-to-noise ratio, such as high-resolution 3D imaging and fibre bundle tracking experiments. FOCM has been specifically designed for ultrafast clearing of thin tissues, and it can clear 300-μm-thick slices within 2 min [15]. FOCM uses high concentration DMSO as a water-miscible polar solvent for rapid tissue clearing. However, high concentration of DMSO would quench the signal of fluorescence proteins [46]. In our experiments, FOCM treatment quenched the fluorescence of many types of fluorescent proteins. Although FOCM has been successfully used for clearing and imaging the slices from Thy1-GFP-M mice [15], we assume that the fluorescent protein expression of this mouse strain is high enough and GFP is more stable, which could compensate for the quenching effect; however, FOCM may not be suitable for clearing samples labelled with a weak signal.

Obtaining large volumes of tissue 3D data in a non-destructive manner is the most attractive aspect of the tissue-clearing technology; however, not all experiments require complete data at the whole-organ scale. In our experiments, we usually used 100-μm to 1-mm-thick slices for clearing and imaging, which was sufficient for the analysis of intact brain areas and fibre tracing. For samples that need to be labelled with dyes, especially those that require immunostaining, some advanced whole-brain staining technologies, such as stochastic electrotransport technology and eFLASH technology, can complete organ-scale immunostaining within a few days [47, 48]; however, these methods require specific equipment and long pre-treatments and are difficult for handling. We reported that AKS was able to preserve a variety of fluorescent dyes well. Thus, samples can be labelled with specific dyes and then cleared with AKS before performing 3D imaging, which can greatly increase the efficiency and flexibility of tissue clearing.

## Conclusions

In summary, AKS has the advantages of ultrafast and simple tissue-clearing ability with wide compatibility. Thus, it can be widely used in routine 3D imaging experiments. It also has the potential to be used in the analysis of samples for time-sensitive experiments. We also believe that our method to actively adjust the fluorescence intensity can be applied to improve the current tissue-clearing agents and the screening process of future tissue-clearing agents.

## Methods

### Experimental animals

The research on animals in this study was approved by the Animal Care and Use Committee of the University of Science and Technology of China. *CRH-cre* mice (B6(Cg)-Crh^tm1(cre)Zjh^/J, JAX stock number 012704) [31], *VIP-cre* mice (Vip^tm1(cre)Zjh^, JAX stock number 010908) [31], Thy1-YFP-H mice (B6.Cg-Tg (Thy1-YFP)HJrs/J, JAX stock number 003782) [33], cre-dependent reporter lines Ai32 mice (B6;129S-Gt (ROSA)26Sor^tm32(CAG-COP4*H134R/EYFP)Hze^/J, JAX stock number 012569) [32], and *Ai14* mice (B6.Cg-Gt (ROSA)26Sor^tm14(CAG-tdTomato)Hze^/J , JAX stock number 007914) [31, 34] were used. *CRH-cre;Ai14* and *CRH-cre;Ai32* mice were generated by crossing *CRH-cre* male mice with *Ai14* or *Ai32* female mice. The mice were kept in stable environmental conditions of temperature (25°C) and humidity (60%), with a 12-h light/dark cycle (8:00 AM to 8:00 PM) and unlimited access to food and water.

### Sample preparation

To obtain the tissue samples, the mice were deeply anaesthetised with chloral hydrate and transcardially perfused with 0.01 M phosphate-buffered saline (PBS) and ice-cold 4% paraformaldehyde (PFA). The brain, testis, kidney, liver, and intestines were collected and incubated in 4% paraformaldehyde at 4°C for 24–48 h for fixation.

Human brain tissues from the China Brain Bank in School of Medicine, Zhejiang University, or those from the Netherlands Brain Bank were used in this study. Both fresh-frozen and formalin-fixed (> 2 years of fixation) tissues from the temporal cortex were obtained. Permission was obtained for a brain autopsy and the use of the material and medical records for research purposes. The fresh-frozen tissues were cut to 0.5–1-cm-thick sections and incubated in 4% paraformaldehyde at 4°C for 48 h for fixation.

After fixation, 100-μm-thick to 1-mm-thick slices were sectioned using a vibration microtome (Leica, VT1200 S). The slices were stored at 4°C in PBS containing 0.05% NaN_3_ for future use, and the storage time was not longer than 1 week.

### Preparation of AKS reagents

To prepare AKS solutions, 20% (vol/vol) DMSO, 40% (vol/vol) TDE, 20% (wt/vol) sorbitol, and 6% (wt/vol, equal to 0.5 M) Tris base were dissolved in ddH2O and stirred at room temperature (25°C). After complete dissolution, the reagents could be stored at room temperature for several months.

### Tissue clearing with AKS

For clearing 0.1–1-mm-thick tissue sections, after washing or labelling, the slices were incubated with AKS at room temperature with shaking at 80 rpm. Typically, the clearing time was 5 min for the 300-μm-thick slices and 60 min for the 1-mm-thick slices. For clearing of hemibrains, the samples were incubated in AKS at room temperature with shaking at 80 rpm in a 50-mL glass centrifuge tube with 30 mL of reagent; typically, the hemibrains became transparent within 8 h. Imaging was recommended to be performed immediately after tissue clearing.

### Tissue optical clearing with other clearing methods

The clearing methods, including CUBIC-1, ScaleSQ (5), and FOCM, followed the protocol as described below.

CUBIC-1. CUBIC-1 reagent was prepared as a mixture of 25% (wt/wt) urea, 25% (wt/wt) N,N,N′,N′-Tetrakis (2-hydroxypropyl) ethylenediamine, and 15% (wt/wt) Triton X-100. The reagents were dissolved in water and stirred at room temperature until well mixed [44].

ScaleSQ (5). ScaleSQ (5) was prepared as a mixture of 9.1 M urea, 22.5% (wt/vol) D-sorbitol, and 5% (wt/vol) Triton X-100. The reagents were dissolved in water, and a microwave oven was used for heating to promote the dissolution of high concentrations of urea [10].

FOCM. FOCM was prepared as a mixture of 30% (wt/vol) urea, 20% (wt/vol) D-sorbitol, and 5% (wt/vol) glycerol dissolved in DMSO. When preparing the reagent, urea and D-sorbitol were dissolved in DMSO and stirred at room temperature until complete dissolution, and then glycerol was added and stirred further [15].

### Immunostaining on the mice and human brain tissues

For staining of 100-μm-thick mouse brain slices, the slices were permeated with PBS containing 0.3% Triton X-100 (PBST) for 30 min and blocked with 5% donkey serum in PBST for 30 min at room temperature. After blocking, the slices were incubated with a primary antibody dilution in PBST at 4°C for 24 h, followed by incubation in a secondary antibody dilution in PBST for 2 h at room temperature. For 3D immunostaining of 300-μm-thick mouse brain slices or 150-μm-thick human brain slices, the entire procedure was performed at room temperature. The slices were permeated with PBST for 1 h and then blocked with 5% donkey serum dilution in PBST for 1 h. After blocking, the slices were incubated with the primary antibody dilution in PBST (containing 0.05% NaN_3_ and 5% donkey serum) for 24 h with shaking at 80 rpm, followed by the incubation with the secondary antibody dilution in PBST (containing 0.05% NaN_3_ and 5% donkey serum) for 24 h at 80 rpm. The antibodies used are listed below.

#### Primary antibody

Rabbit polyclonal antibody to GFAP (DAKO, Z0334) was used for labelling astrocytes, at the concentration of 1:1000.

#### Secondary antibodies

DyLight 405-conjugated donkey IgG (711-475-152), Alexa 488-conjugated donkey IgG (711-545-152), Alexa 594-conjugated donkey IgG (711-585-152), and Alexa 647-conjugated donkey IgG (711-605-152); all of the secondary antibodies were purchased from Jackson ImmunoResearch, and their working dilution was 1:200.

### NeuroTrace staining

For NeuroTrace staining, the slices were permeated with PBST for 30 min and then incubated in NeuroTrace 435/455 solution (Life Technologies, Cat. No. N-21479, 1:50 dilution in PBST) for 30 min at room temperature.

### Fluoro-Gold, RetroBeads, and virus injection

For labelling of Fluoro-Gold and RetroBeads, adult C57BL/6 or Thy1-YFP mice were deeply anaesthetized with isoflurane and fixed in the stereotaxic. An electrode containing 100 nL of Fluoro-Gold or RetroBeads (Lumafluor, Red-1X) was implanted unilaterally into the corpus callosum (right hemisphere, AP, −1.9 mm; ML, 0.8 mm; DV, −0.9 mm) and injected. The mice were killed for further experiments after 48 h. For labelling of mCherry, adult *Vip-cre* mice were deeply anaesthetized with isoflurane and fixed in the stereotaxic. An electrode containing 200 nL of rAAV-CAG-DIO-mCherry (BrainVTA) was implanted unilaterally into the suprachiasmatic nucleus (SCN, right hemisphere, AP, −0.3 mm; ML, 0.2mm; DV, −5.5 mm) and injected. The mice were killed for further experiments after 3 weeks to examine mCherry expression.

### Biocytin labelling

We used 300-μm-thick acute brain slices for biocytin labelling. Acute brain slices were prepared as described before [49]. An electrode with internal solutions (130 mM K-gluconate, 10 mM HEPES, 0.1 mM EGTA, 10 mM NaCl, 2 mM MgCl_2_, 10 mM phosphocreatine (tris), 4 mM Mg-ATP, and 0.3 mM Na-GTP; 290 mOsm/L; pH 7.2) containing 0.1% Alexa 488-conjugated biocytin (Thermo Scientific, A12924) was inserted into the slices, and the internal solution was infused into the tissue; after 10 min, the slices were collected and fixed with 4% PFA overnight. Next, the slices were washed with PBS and stored at 4°C in PBS containing 0.05% NaN_3_ for future use.

### Thioflavin-S staining

After immunofluorescence staining, 150-μm-thick slices from Alzheimer’s disease (AD) patient (Braak VI) temporal cortex were incubated in 0.5% thioflavin-S dilution in 50% ethanol for 10 min and then washed with 50% ethanol for 10 min. Next, the slices were washed with PBST for 10 min to remove residual ethanol and then stored at 4°C in PBS before imaging or clearing.

### Mounting processing

To mount tissue slices thinner than 100 μm, the slices were directly mounted on a positively charged adhesion microscope slide and air dried for 30 min to let the section adhere to the slide and then washed in PBS for 30 min for rehydration. Next, the mounted slices were treated with a clearing reagent for a specific time and sealed with cover glass for imaging. To mount tissue slices thicker than 100 μm, an agarose holder (25 ×25 mm with a 10 × 8 mm hole) was obtained by cutting 3% agarose into appropriate thickness (equal to tissue slice) using vibration microtome and then glued to a glass coverslip (25 × 50 mm). The holder was filled with AKS reagent and the cleared tissue was placed into the holder. Another glass coverslip (23 × 23 mm) was placed on the holder to block the tissue from the air (Additional file 6: Fig. S6).

### Imaging

Three microscope systems were used for the experiments in this study:

i. Olympus two-photon microscope system (FVMPE-RS) equipped with a 10× water-immersion objective lens (UMPLFLN10X; numerical aperture (NA), 0.30; working distance (WD), 3.5 mm) and a 25× water-immersion objective lens (XLPLN25XWML; NA, 1.05; WD, 2.0 mm). We used 920 nm and 1040 nm for the excitation of YFP and tdTomato, respectively.
ii. Olympus confocal microscope system (FV1200MPE) equipped with a 10× air objective lens (UPLSAPO10X; NA, 0.4; WD, 3.1 mm), a 20× air objective lens (UCPLFLN20X; NA, 0.7; WD, 0.8 mm), and a 60× oil immersion objective lens (PLAPON60X; NA, 1.4; WD,0.12 mm). We used 405 nm, 488 nm, 594 nm, and 633 nm 1p lasers.
iii. Zeiss confocal microscope system (LSM880) equipped with a 10× air objective lens (Plan-Apochromat 10X/0.45 M27), a 25× glycerol-immersion objective lens (LCI Plan-Neofluar 25x/0.8 Imm Korr DIC M27), a 20× air objective lens (Plan-pochromat 20x/0.8 M27), and a 63× oil immersion objective lens (Plan-Apochromat 63x/1.4 Oil DIC M27). We used 488 nm and 633 nm 1p lasers.

### Image processing and statistical analysis

3D reconstruction of the images was performed by using Imaris software (Bitplane), and all the data were presented as mean ± standard error of mean (mean ±SEM). To analyse the morphology changes after AKS treatment (Fig. 1B), a one-way analysis of variance (ANOVA) was used, followed by Bonferroni’s multiple comparison tests. To compare the optical transmittance before and after the AKS treatment in Fig. 1D, a paired *t*-test was used. For fluorescence quenching analysis (Fig. 2C), the samples were imaged at the same position with the same imaging parameters at a certain time point. The average intensity was obtained by using ImageJ software, and a one-way analysis of variance (ANOVA) was used, followed by Bonferroni’s multiple comparison tests.

## Supplementary Information

**Additional file 1:** **Figure S1.** Measurement of the fluorescence intensity of YFP before and after AKS treatment. Specifically, 20-μm- thick slices from Thy1-YFP mice brains were embedded on an adhesion microscope glassed, and 3D imaging of a single YFP-labelled neuron was performed using confocal microscopy with the same imaging parameters before and after AKS treatment. **(A)** The example image shows the fluorescence intensity of a single YFP-labelled neuron before and after AKS treatment. Representative of n = 7 neurons (Scale bar: 10 μm). **(B)** Fluorescence corresponding to the same neurons indicated by the dash line in A. Representative of n = 7 neurons. All values are included in Additional file 11: Table S5 **(C)** Analysis of the fluorescence of the background shows that the background is unchanged before and after AKS treatment (n = 7 background area). All values are included in Additional file 11: Table S6. **(D)** Analysis of the fluorescence of the YFP signal, showing that the fluorescence of YFP was enhanced after AKS treatment (*n* = 7 YFP-labelled neurons). All values are included in Additional file 11: Table S7. Statistical significance (**** *p* < 0.0001) was assessed by two-tailed Student’s t-test.

**Additional file 2:** **Figure S2.** The fluorescence preservation before and after 10 min, 24 h, 7 days, and 14 days of treatment with AKS. We tested four fluorescent proteins and nine fluorescent dyes, using the same imaging parameters to observe the same location of the slices at all detection time points. We found that AKS treatment for 10 min only reduced the fluorescence of Dylight405, and other fluorescence signals were well preserved. However, after two weeks of AKS treatment, the tdTomato signal and Retro-Beads signal also weakened, but imaging could still be performed. Representative of n = 3 slices each conditioning. (Scale bar: 50 μm).

**Additional file 3:** **Figure S3.** Analysis of the fluorescence quench of ChR2-YFP before and after AKS treatment at the same imaging parameters. **(A)** Time-stack images showing the photobleaching effect of ChR2-YFP before and after AKS treatment around imaging time at identical laser power. Representative of n = 3 slices (Scale bar: 20 μm). **(B)** Analysis of the relative mean fluorescence intensity around time-series imaging of **A** (n = 3 slices). All values are included in Additional file 11: Table S8.

**Additional file 4:** **Figure S4.** 3D imaging of peripheral tissues cleared with AKS using two-photon microscopy. Tissue sections from 3-month-old mouse testis (300-μm-thick), intestines, liver (200-μm-thick), and kidney (200-μm-thick) were permeated in PBS containing 0.3% Triton X-100 (PBST) for three hours at room temperature, and then washed with PBST for three times, each time for 1 h. After washing, the tissues were stained with a solution containing DAPI and lectin-Dylight594 (DAPI was used at 1:1000 and lectin-Dylight594 (VECTOR laboratories, DL-1177-1) was used at 1:500 diluted in PBST contain 0.05% NaN_3_), and staining was processed at 37°C for 24 h. Then the sample was washed three times with PBST, each time for one hour. After washing, the tissues were imaged in PBST using two-photon microscopy. We used an 800 nm laser for excitation of DAPI and Dylight594. After the imaging, the tissues were cleared in AKS for 1 h at room temperature, and then imaged with the same imaging parameters used before clearing. We found that AKS treatment increased the imaging depth of all these tissues, which indicated that AKS could be used for 3D imaging of peripheral tissues. It should be noticed that after AKS treatment, DAPI was visible in the entire imaging range, but lectin-Dylight594 only appeared on the surface of the liver and kidney slices. We assume that it may be due to our insufficient staining time or insufficient amount of dye, which have resulted in only the surface staining. Representative of n = 3 samples of each tissue.

**Additional file 5:** **Figure S5.** Analysis of the morphology change of 300-μm-thick human brain slices after 10 min of AKS treatment. **(A)** Image of three 300-μm-thick human brain slices before and after AKS treatment. **(B)** Analysis of the morphology change before and after 10 min of AKS treatment (n = 3 slice). The tissue linear expansion was 96.33 ± 2.06% (mean ± SEM) after 10 min AKS treatment and had no significant difference compared with PBS (*p* >0.05). Statistical significance was assessed by two-tailed Student’s *t*-test. All values are included in Additional file 11: Table S9.

**Additional file 6:** **Figure S6.** Schematic diagram of sample mounting procedure after optical imaging.

**Additional file 7:** **Movie S1.** High-resolution three-dimensional imaging of *CRH-cre;Ai32* mice central amygdala.

**Additional file 8:** **Movie S2.** 3D visualisations of tdTomato-labelled CRH neurons (red) in *CRH-cre;Ai14* mice paraventricular nucleus of the hypothalamus.

**Additional file 9:** **Movie S3.** 3D visualisation of Thy1-YFP mouse brain slices immunostained for the astrocyte marker. YFP, green; astrocyte, red.

**Additional file 10:** **Movie S4.** 3D visualisation of thioflavin-S labelled neuritic plaques (green) and GFAP labelled astrocytes (red) in human brain samples with Alzheimer’s disease.

**Additional file 11:** The individual raw data values of figures and Additional files for number of replicates ≤ 6. All the data are cited in the figure legend.

## Data Availability

All data generated or analysed during this study are included in this published article and its supplementary information files.

## Abbreviations

3D: Three-dimensional
AD: Alzheimer’s disease
AKS: Alkaline solution
CRH: Corticotropin-releasing factor
DAPI: 4′,6-Diamidino-2-phenylindole
DMSO: Dimethyl sulfoxide
EYFP: Enhanced yellow fluorescent protein
GFAP: Glial fibrillary acidic protein
LSCM: Laser scanning confocal microscope
MOST: Micro-optical sectioning tomography
RI: Refractive index
TDE: 2,2′-Thiodiethanol
YFP: Yellow fluorescent protein
