# Supplementary figures and images for: A method for ultrafast tissue clearing that preserves fluorescence for multimodal and longitudinal brain imaging
Source: BMC Biol. 2022 Mar 29;20:77. doi: 10.1186/s12915-022-01275-6 (PMC8966190; doi:10.1186/s12915-022-01275-6)

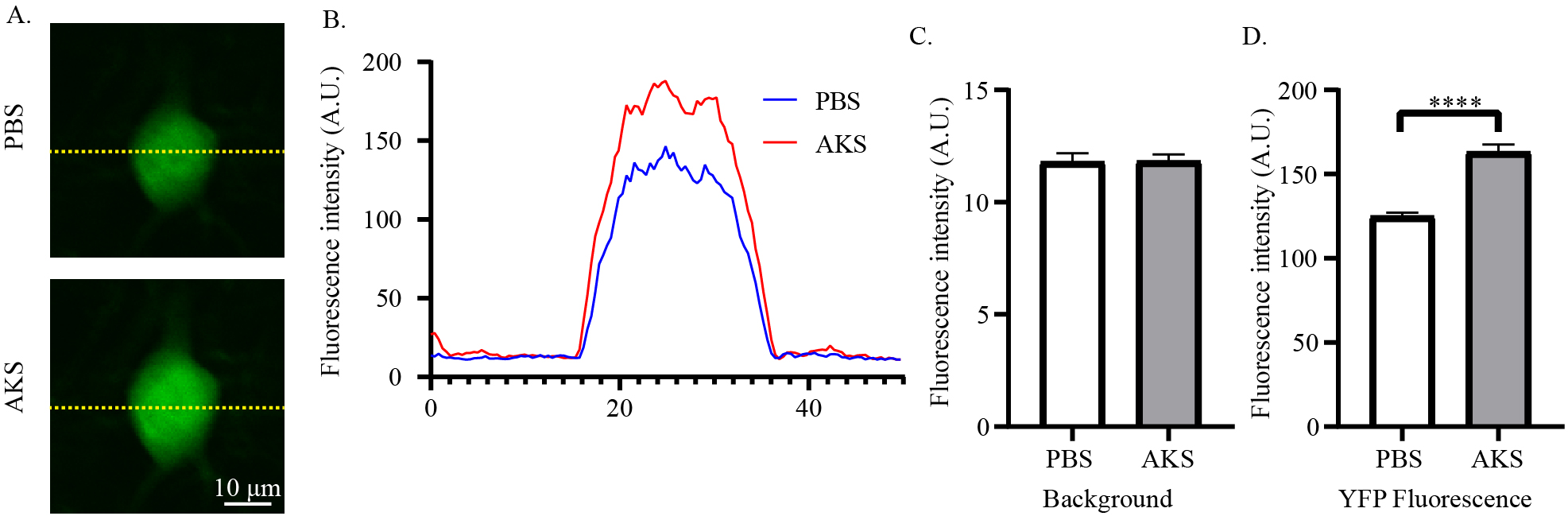

Supplement: Supplementary file 1 — Additional file 1: Figure S1. Measurement of the fluorescence intensity of YFP before and after AKS treatment. Specifically, 20-μm- thick slices from Thy1-YFP mice brains were embedded on an adhesion microscope glassed, and 3D imaging of a single YFP-labelled neuron was performed using confocal microscopy with the same imaging parameters before and after AKS treatment. (A) The example image shows the fluorescence intensity of a single YFP-labelled neuron before and after AKS treatment. Representative of n = 7 neurons (Scale bar: 10 μm). (B) Fluorescence corresponding to the same neurons indicated by the dash line in A. Representative of n = 7 neurons. All values are included in Additional file 11: Table S5 (C) Analysis of the fluorescence of the background shows that the background is unchanged before and after AKS treatment (n = 7 background area). All values are included in Additional file 11: Table S6. (D) Analysis of the fluorescence of the YFP signal, showing that the fluorescence of YFP was enhanced after AKS treatment (n = 7 YFP-labelled neurons). All values are included in Additional file 11: Table S7. Statistical significance (**** p < 0.0001) was assessed by two-tailed Student’s t-test. [file 12915_2022_1275_MOESM1_ESM.jpg]

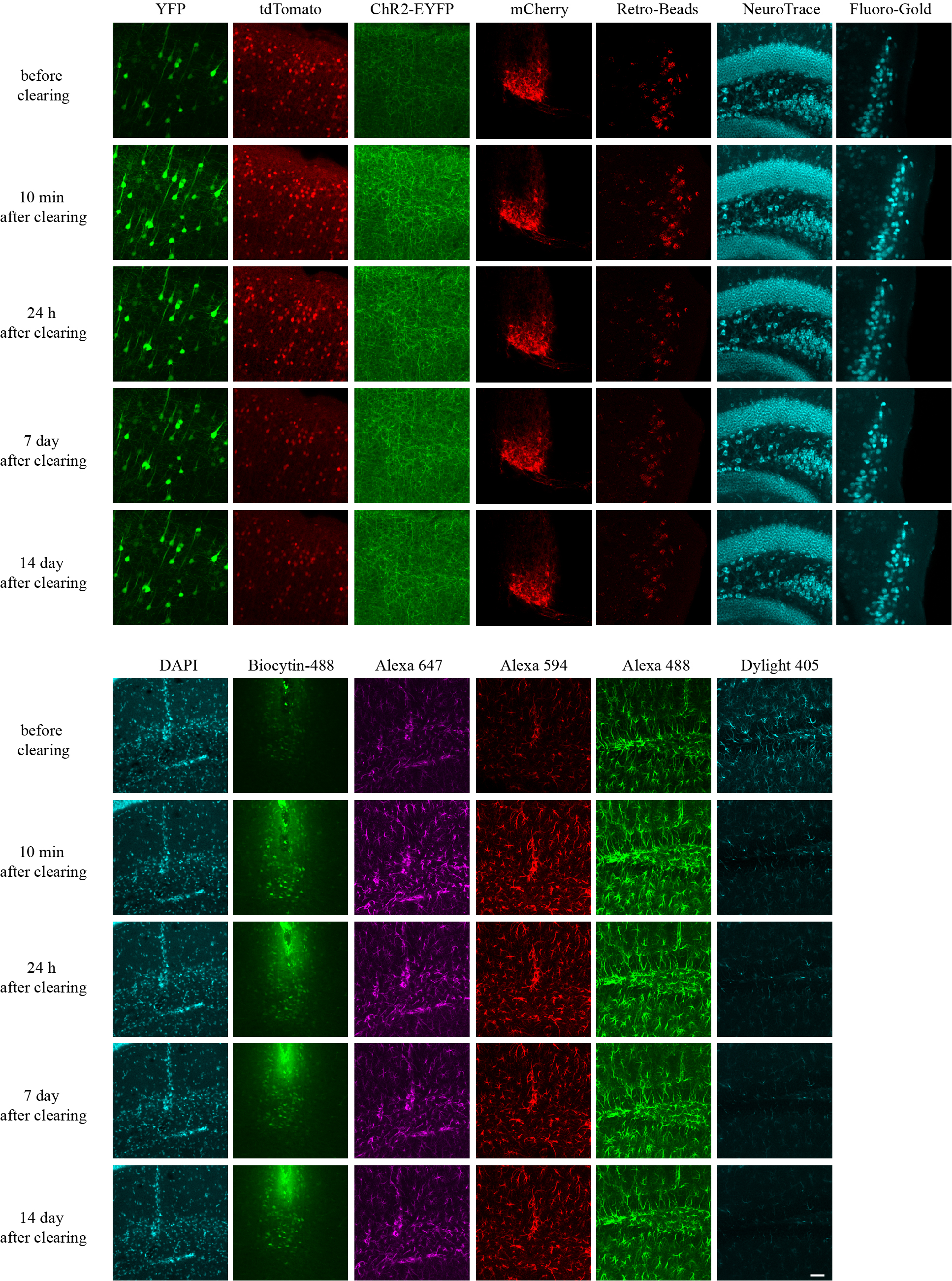

Supplement: Supplementary file 2 — Additional file 2: Figure S2. The fluorescence preservation before and after 10 min, 24 h, 7 days, and 14 days of treatment with AKS. We tested four fluorescent proteins and nine fluorescent dyes, using the same imaging parameters to observe the same location of the slices at all detection time points. We found that AKS treatment for 10 min only reduced the fluorescence of Dylight405, and other fluorescence signals were well preserved. However, after two weeks of AKS treatment, the tdTomato signal and Retro-Beads signal also weakened, but imaging could still be performed. Representative of n = 3 slices each conditioning. (Scale bar: 50 μm). [file 12915_2022_1275_MOESM2_ESM.jpg]

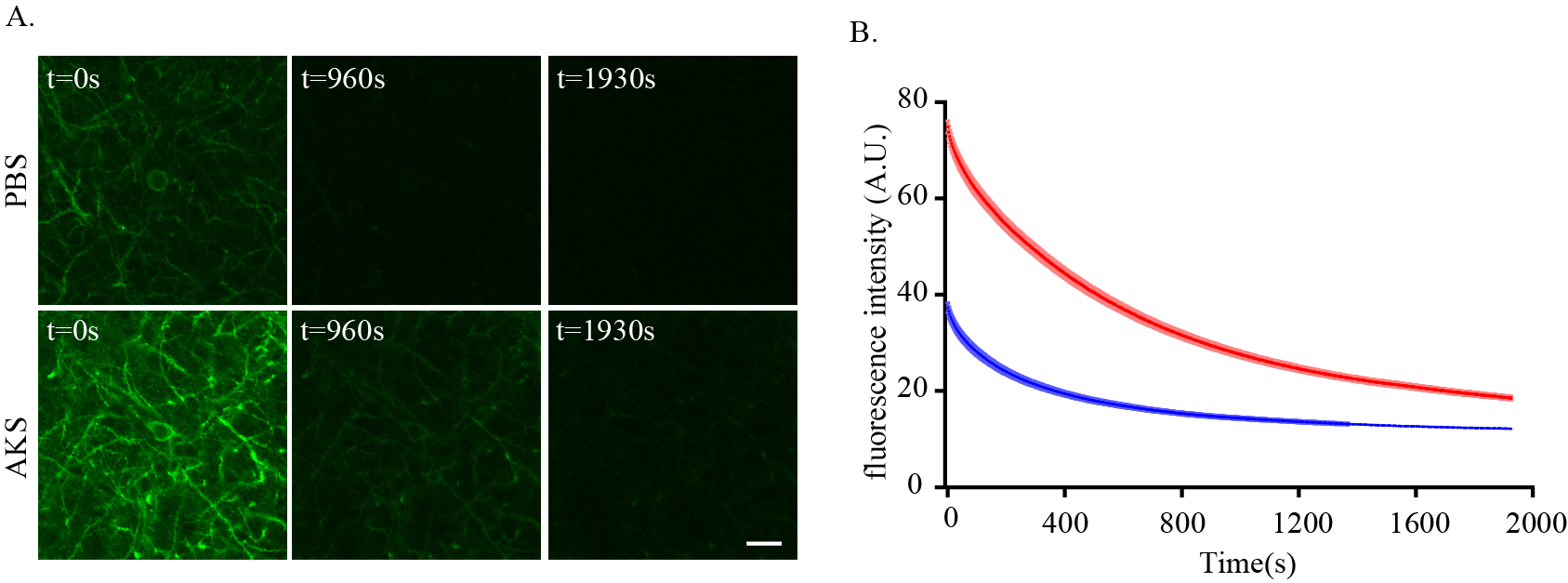

Supplement: Supplementary file 3 — Additional file 3: Figure S3. Analysis of the fluorescence quench of ChR2-YFP before and after AKS treatment at the same imaging parameters. (A) Time-stack images showing the photobleaching effect of ChR2-YFP before and after AKS treatment around imaging time at identical laser power. Representative of n = 3 slices (Scale bar: 20 μm). (B) Analysis of the relative mean fluorescence intensity around time-series imaging of A (n = 3 slices). All values are included in Additional file 11: Table S8. [file 12915_2022_1275_MOESM3_ESM.jpg]

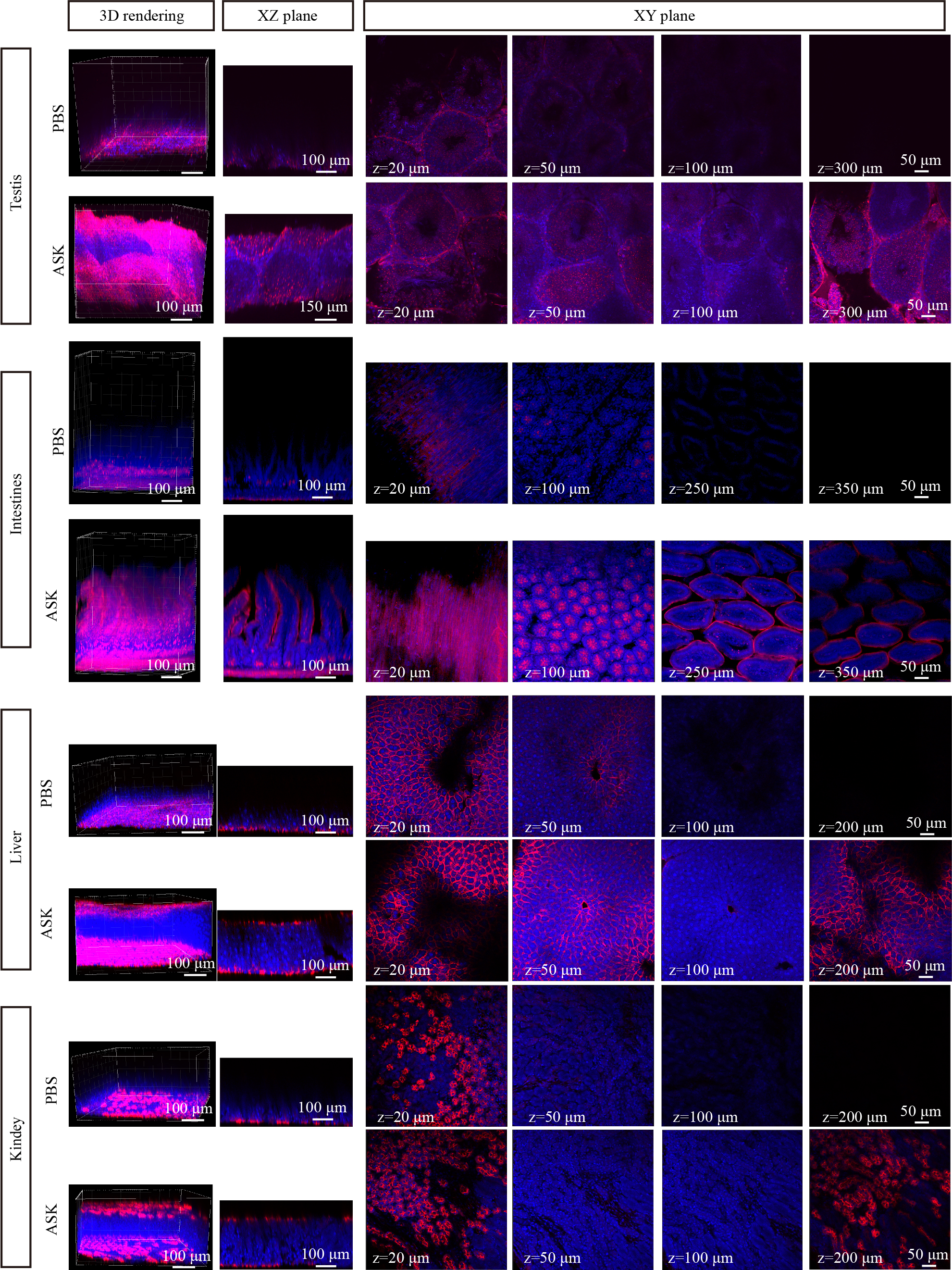

Supplement: Supplementary file 4 — Additional file 4: Figure S4. 3D imaging of peripheral tissues cleared with AKS using two-photon microscopy. Tissue sections from 3-month-old mouse testis (300-μm-thick), intestines, liver (200-μm-thick), and kidney (200-μm-thick) were permeated in PBS containing 0.3% Triton X-100 (PBST) for three hours at room temperature, and then washed with PBST for three times, each time for 1 h. After washing, the tissues were stained with a solution containing DAPI and lectin-Dylight594 (DAPI was used at 1:1000 and lectin-Dylight594 (VECTOR laboratories, DL-1177-1) was used at 1:500 diluted in PBST contain 0.05% NaN3), and staining was processed at 37°C for 24 h. Then the sample was washed three times with PBST, each time for one hour. After washing, the tissues were imaged in PBST using two-photon microscopy. We used an 800 nm laser for excitation of DAPI and Dylight594. After the imaging, the tissues were cleared in AKS for 1 h at room temperature, and then imaged with the same imaging parameters used before clearing. We found that AKS treatment increased the imaging depth of all these tissues, which indicated that AKS could be used for 3D imaging of peripheral tissues. It should be noticed that after AKS treatment, DAPI was visible in the entire imaging range, but lectin-Dylight594 only appeared on the surface of the liver and kidney slices. We assume that it may be due to our insufficient staining time or insufficient amount of dye, which have resulted in only the surface staining. Representative of n = 3 samples of each tissue. [file 12915_2022_1275_MOESM4_ESM.jpg]

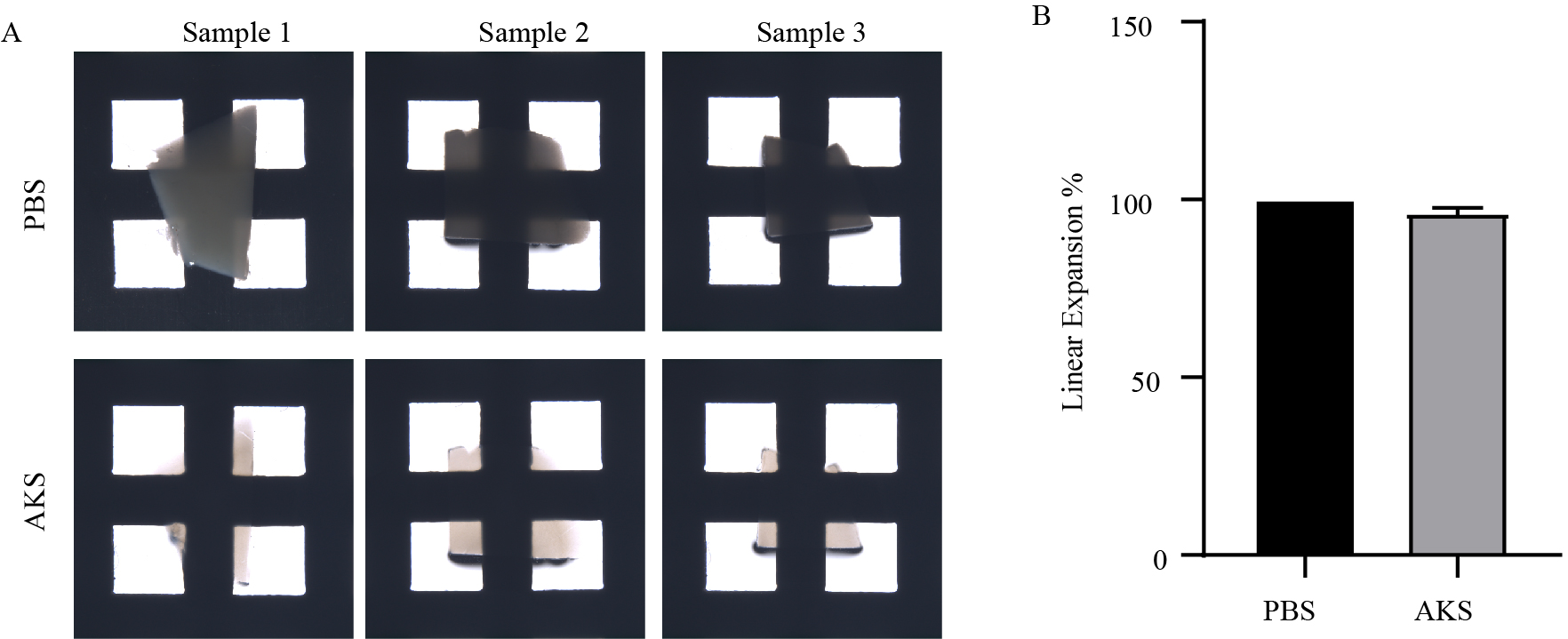

Supplement: Supplementary file 5 — Additional file 5: Figure S5. Analysis of the morphology change of 300-μm-thick human brain slices after 10 min of AKS treatment. (A) Image of three 300-μm-thick human brain slices before and after AKS treatment. (B) Analysis of the morphology change before and after 10 min of AKS treatment (n = 3 slice). The tissue linear expansion was 96.33 ± 2.06% (mean ± SEM) after 10 min AKS treatment and had no significant difference compared with PBS (p >0.05). Statistical significance was assessed by two-tailed Student’s t-test. All values are included in Additional file 11: Table S9. [file 12915_2022_1275_MOESM5_ESM.jpg]

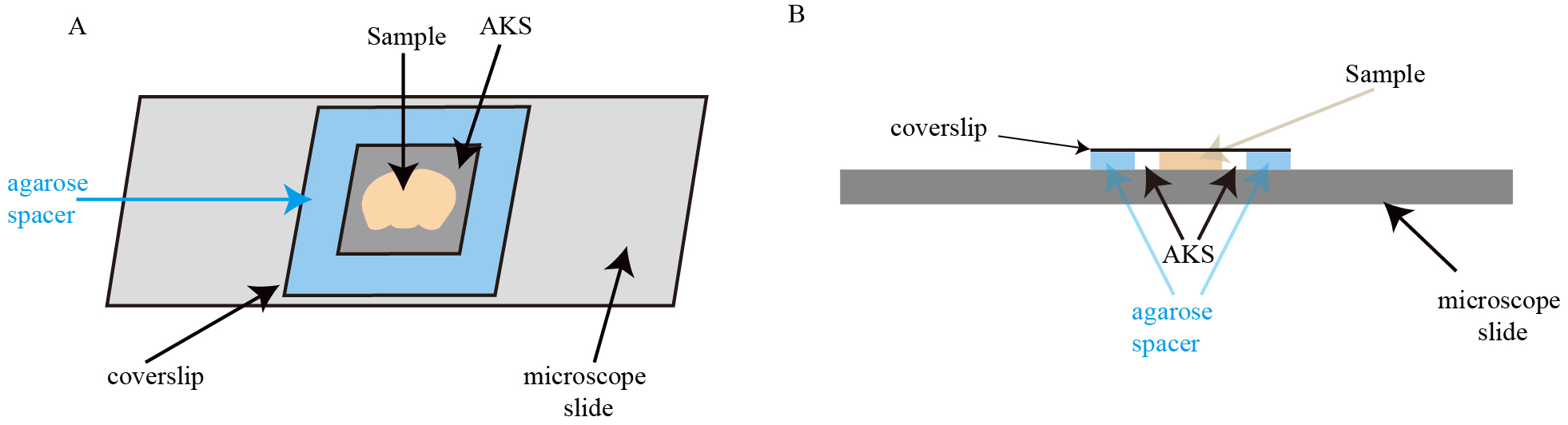

Supplement: Supplementary file 6 — Additional file 6: Figure S6. Schematic diagram of sample mounting procedure after optical imaging. [file 12915_2022_1275_MOESM6_ESM.jpg]
